# Giant coronary artery fistula complicated with coronary artery aneurysm and acute myocardial infarction: a case report

**DOI:** 10.1186/s12872-020-01415-2

**Published:** 2020-03-14

**Authors:** Zhuoxuan Yang, Liang Zhang, Jin Gao, Jingang Cui, Jiansong Yuan, Shengwen Liu, Yue Zhou, Shubin Qiao

**Affiliations:** 1 Department of Cardiology, Chinese Academy of Medical Sciences and Peking Union Medical College Fuwai Hospital, North Lishi Road, Beijing, 100037 China; 2 Cardiology Department, Yuncheng Central Hospital, City, Shanxi province, Yuncheng, 044000 China; 3 Cardiology Surgery Department, Yuncheng Central Hospital, City, Shanxi province, Yuncheng, 044000 China

**Keywords:** Coronary artery fistula, Coronary artery aneurysm, Acute myocardial infarction

## Abstract

**Background:**

Coronary artery fistula (CAF) is an abnormal connection between a coronary artery and either a cardiac chamber or the great vessels. Although most patients are asymptomatic, potential complications such as heart failure, angina pectoris or acute myocardial infarction can be fatal.

**Case presentation:**

We present here a 62-year-old man diagnosed with giant coronary artery fistula complicated with gross coronary artery aneurysm and acute myocardial infarction. He underwent intravenous thrombolysis treatment at a local hospital, coronary angiography at a regional hospital and complex surgery at a national centre for cardiovascular disease. The patient had no major adverse cardiac events during the 3-year follow-up.

**Conclusion:**

Early diagnosis of CAF patients and an appropriate treatment plan are the key factors for avoiding serious complications. Because of the rare incidence of this disease, it is necessary to discover and discuss management strategies, including medical management, percutaneous interventions or surgical treatment, for a successful outcome.

## Background

Coronary artery fistula (CAF) is an abnormal connection between a coronary artery and either a cardiac chamber or the great vessels [1], which was first described by Krause in 1865 [2]. It can be congenital or acquired and has an estimated prevalence of 0.002% in the general population but the prevalence is 0.25–0.5% in patients who undergo coronary angiography [3]. Most CAF patients were asymptomatic and identified by physical examination.

We present herein a rare case of coronary artery fistula complicated with coronary artery aneurysm and acute myocardial infarction.

## Case presentation

**Fig. 1:**
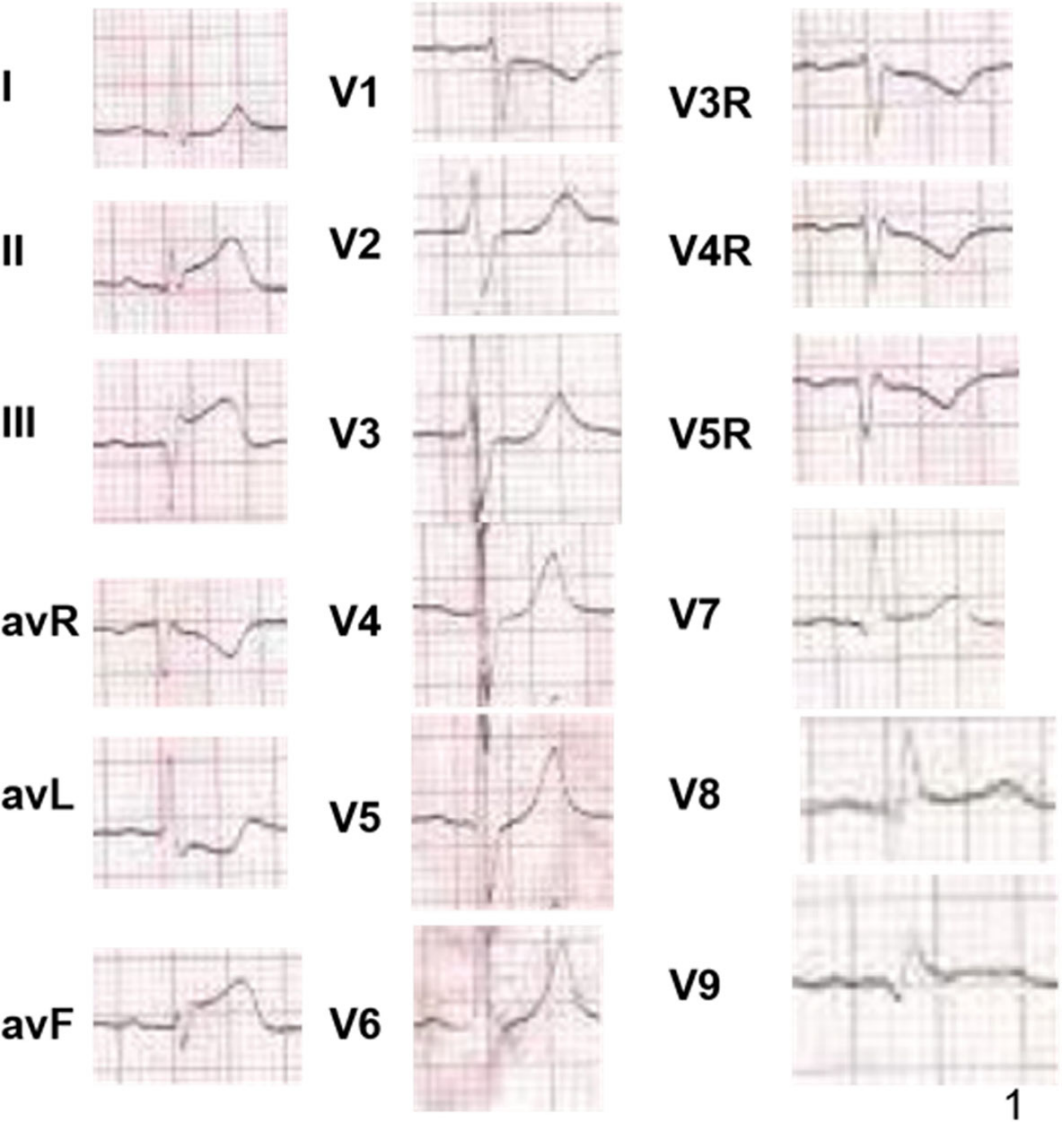
ECG when patient admitted in the local hospital

A 62-year-old hypertentive man was admitted to a local hospital for persistent chest pain lasting 1 h. The patient said he had a heart murmur during a physical examination at age 8 but was misdiagnosed with mitral insufficiency, so he was not further treated. The electrocardiograph (ECG) demonstrated a significant ST-segment elevation in II,III,avF and V_7–9_ (Fig. 1). A diagnosis of acute inferior-posterior myocardial infarction was made. The patient was given 300 mg aspirin,300 mg clopidogrel and then intravenous thrombolysis treatment (Reteplase 18 mg*2). However he still had chest pain and ST-segment elevation 2 h after treatment. Then, he was transferred to a regional percutaneous coronary intervention (PCI)-capable hospital.

At admission, his blood pressure was 126/62 mmHg, and his heart rate was regular at 122 beats/min without cardiac murmurs. There was no systemic or pulmonary oedema. The cTNI was 6.64 mmol/L, NT-proBNP was 664.8 pg/mL, and D-dimer was 37,180 ng/mL.

**Fig. 2:**
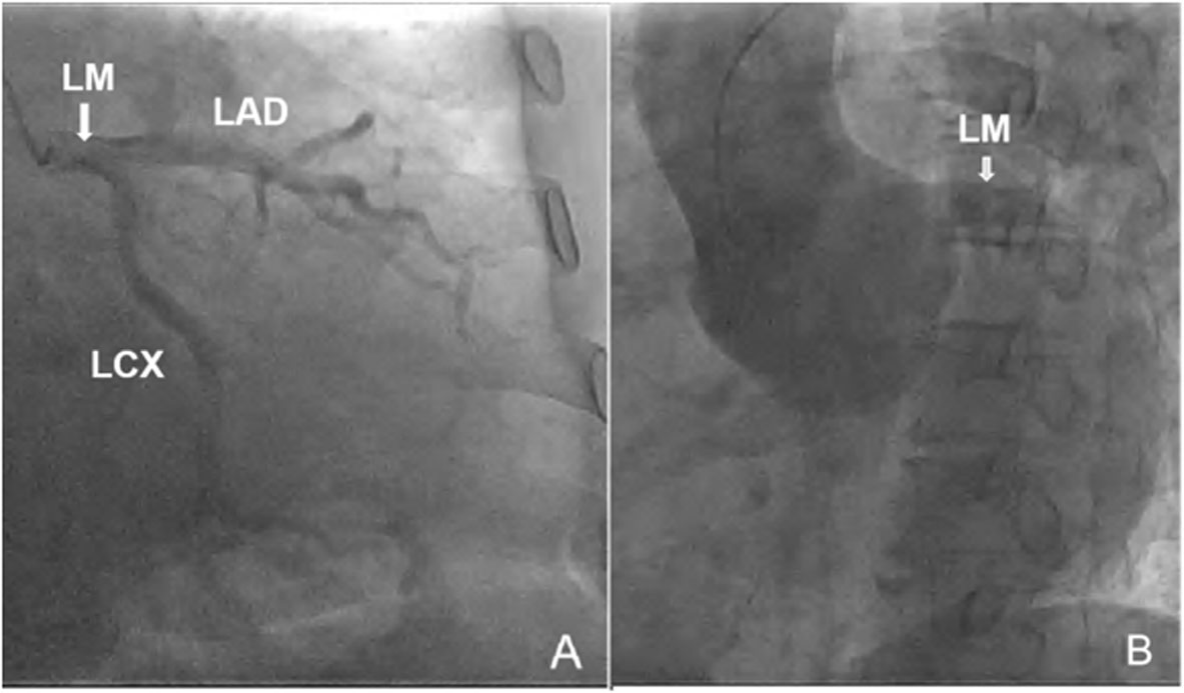
Coronary angiography showing the LM, LAD and LCX (**a**). The ostia of the RCA couldn’t be observed. (**b**)

Emergence coronary angiography was performed and showed a 50–60% stenosis in the middle left anterior descending artery (mLAD), and the left circumflex artery (LCX) was normal (Fig. 2a). When they tried to perform right coronary artery (RCA) angiography, the catheter could not enter the RCA. Ultimately, the physicians were still unable to observe the ostia of the RCA (Fig. 2b).

**Fig. 3:**
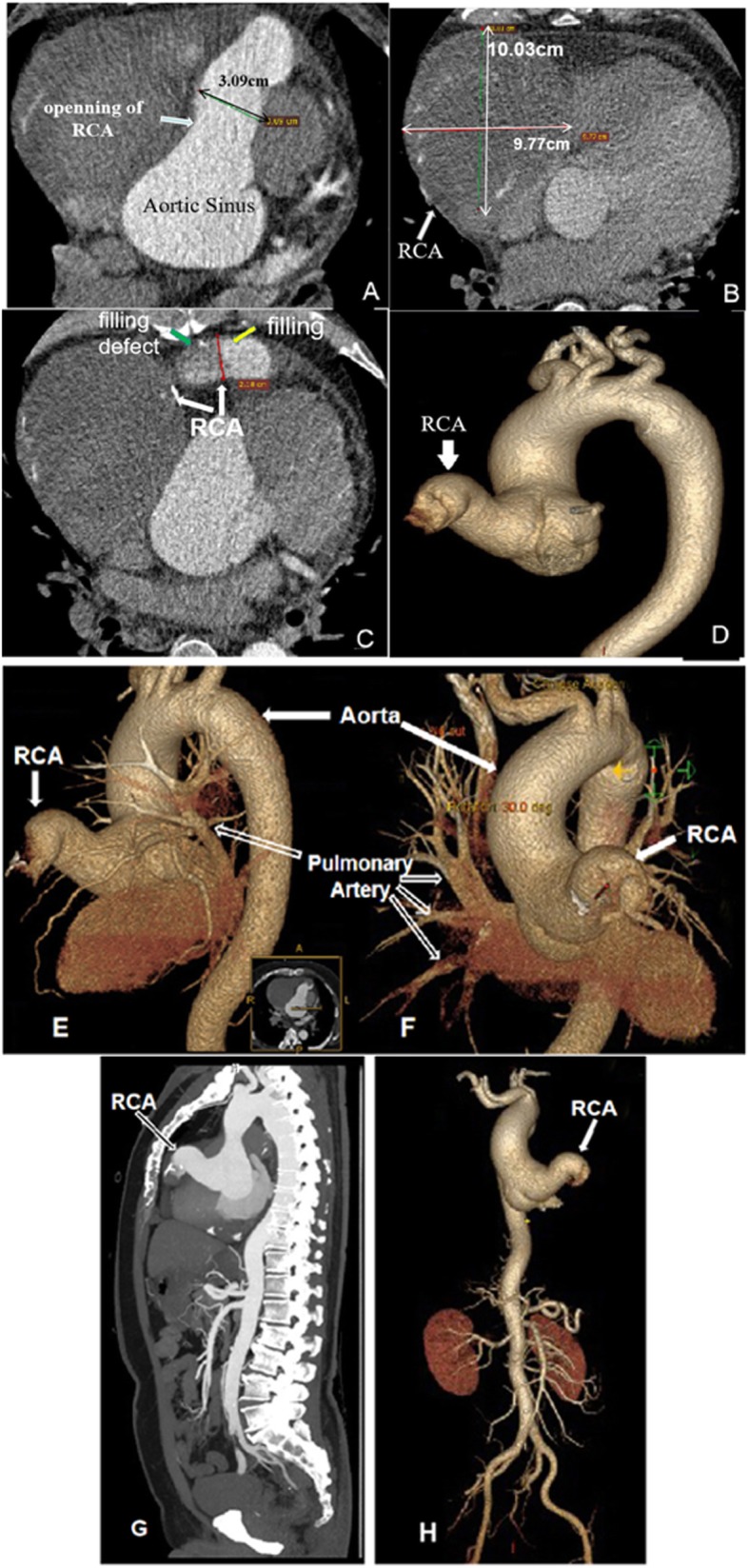
**a-d** Coronary computed tomography. CT (coronary view) showing origin of the dilated coronary artery from the aortic sinus and the opening of RCA is 3.09 cm (**a)**; the widest part of RCA is more than 10 cm (**b**); the thrombus blocked the RCA (**c**) and 3D reconstruction of the heart showing dilated RCA originated from the ascending aorta (**d**). (**e**-**h**) Coronary computed tomography. 3D reconstruction of the heart and great vessels.

Echocardiography revealed a giant right coronary artery- right ventricle fistula. A computed tomography angiography (CTA) scan 7 days after admission showed that the dilated RCA opening was approximately 30 mm (Fig. 3a), and the widest segment was about 97 mm (Fig. 3b). The thrombus blocked the artery flow, and the contrast medium filling in the distal region was defective (Fig. 3c). 3D reconstruction of the heart showed a dilated and tortuous RCA originating from the ascending aorta and traversing through the right front the heart, but its development stopped because the flow was blocked (Fig. 3d). The 3D reconstruction of the heart and great vessels showed that the diameter of the RCA was almost equal to that of the descending aorta (Fig. 3e-h).

Therefore, a diagnosis of congenital right coronary artery-right ventricular fistula complicated with coronary artery aneurysm and acute myocardial infarction was made. The patient had discovered a cardiac murmur at age 8 but had been was asymptomatic for 62 years before the acute myocardial infarction occurred. After tortuous treatments, including intravenous thrombolysis treatment at a local hospital, coronary angiography was performed at a regional hospital. He was ultimately transferred to our hospital for complex surgical treatment.

After 1 month of medication, he was admitted to our hospital and underwent surgery for coronary artery fistula repair + coronary aneurysm resection + coronary aneurysm thrombectomy + aortic sinus plasty. During the operation, the RCA was tortuously dilated, with the widest point > 100 mm located in the right atrioventricular groove at 120 mm in length, causing compression on the right lung. Many red thrombi intermingled with white blood clots in the lumen were observed after the RCA was cut open. The dilated segment of the RCA was cut and removed, followed by opening of the RCA and the long axis to the aortic root. The ostia of the RCA was expanded approximately 30 mm, with a longitudinal suture line, cutting and forming the right coronary sinus. The distal RCA in the right ventricle was stitched. The right crown was small, the lumen was approximately 1 mm, and it was not treated.

**Fig. 4:**
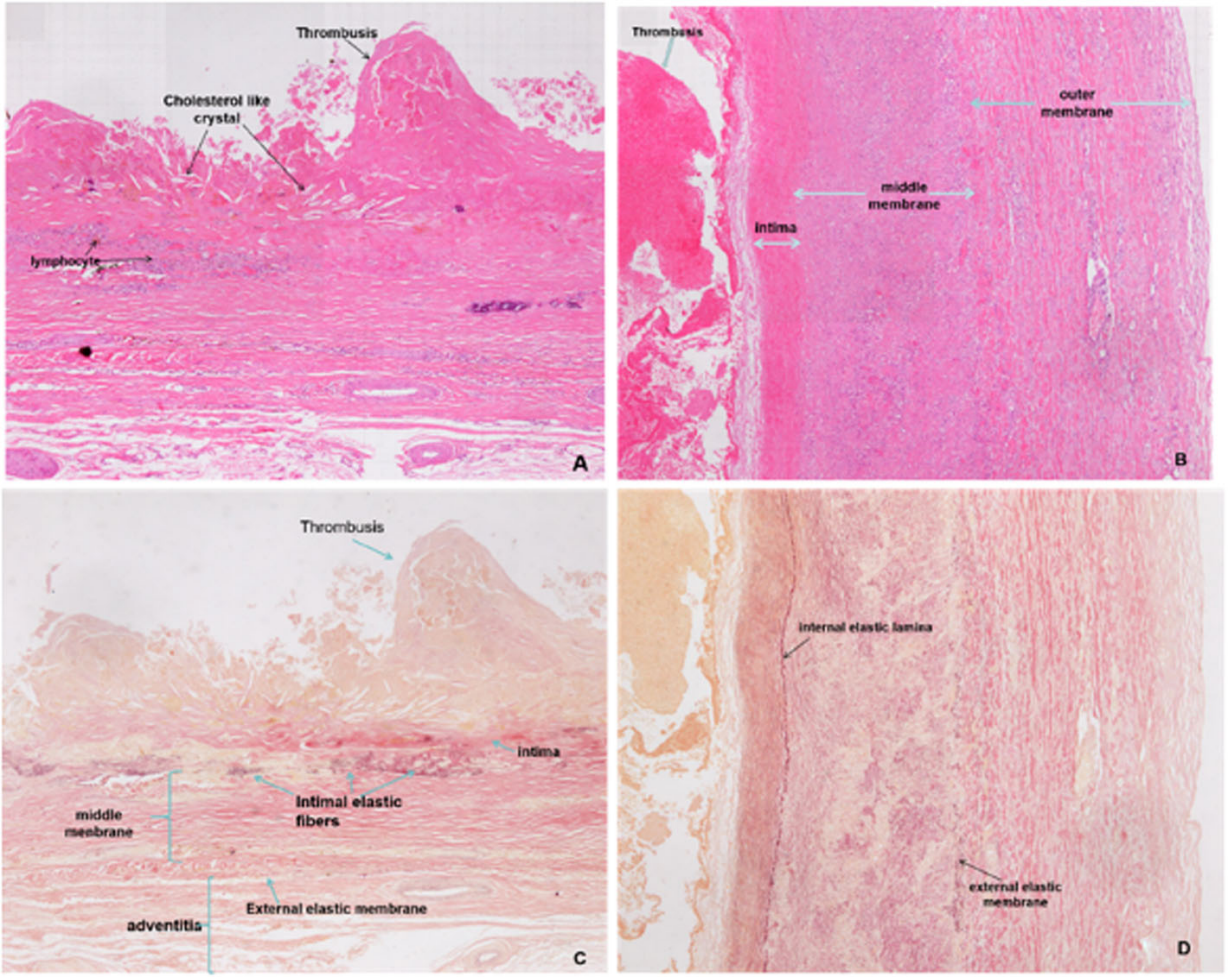
**a-d** Pathological images. (**a)** showed the formation of atherosclerotic plaque in the intima and partial organization of mural thrombus, lymphocyte infiltration was observed in the glossy dilated segment. The three layers in the relative normal coronary artery wall segment (**b**). The middle membrane of the artery was hypertrophic and atrophic, with partial replacement of the collagen fibers and fibrous thickening of the membrane (**c**). The internal elastic lamina and external elastic membrane in the relative normal coronary artery wall segment (**d**)

The occluded part of the middle of the RCA was sent for pathological examination. Images (Fig. 4a-d) show the formation of atherosclerotic plaques in the intima and partial organization of the mural thrombus, and lymphocyte infiltration was observed in the grossly dilated segment (Fig. 4a). The three layers in the relatively normal coronary artery wall segment are shown in Fig. 4b. The middle membrane of the artery was hypertrophic and atrophic, with partial replacement of collagen fibres and fibrous thickening of the outer membrane (Fig. 4c). The internal elastic lamina and external elastic membrane in the relatively normal coronary artery wall segment are shown in Fig. 4d.

The patient was satisfied with his treatment and outcome. He had taken aspirin 100 mg once a day, atorvastatin 20 mg once a day, isosorbide 20 mg twice a day, and atenolol 25 mg three times a day. After a 3-year follow-up, he had no angina pectoris, myocardial infarction or heart failure.

## Discussion and conclusion

Congenital heart disease is the primary reason for CAFs, which may be caused by infection in early embryonic development or genetic factors [4]. In early foetal development, the intramyocardial trabecular sinusoids freely connect the heart cavity with the coronary veins and arteries, but later, they are compressed into tiny tubes, forming Thebesian veins. If the spaces persist, CAFs can be formed [5]. Trauma and invasive procedures such as endomyocardial biopsy are the other reasons for CAFs.

Approximately 50% of CAFs originate from the right coronary artery, followed by the left anterior descending branch, and CAFs originating from the circumflex branch or left main artery are rare. Over 90% of CAFs terminate into the right heart (approximately 41% in the right ventricle, 26% in the right atrium), and approximately 3% terminate into the left heart [2].

Approximately 17% of patients with CAFs have complications with coronary artery aneurysm [3]. When the diameter of the aneurysm is ≥8.0 mm, it is called a giant coronary artery aneurysm [6]. This may be because the pressure in the left heart is higher than that in the right heart. A long-term left-to-right shunt leads to increased pressure and blood flow in the coronary artery, which leads to the destruction of the middle elastic fibre layer in the inner wall of the blood vessel. Then, vascular compliance decreases, resulting in dilation and formation of aneurysms. Atry and Turkt [6] found that the degree of fistula dilation is not necessarily proportional to blood flow but is significantly correlated with mortality. In this case, the patient was asymptomatic before the event. Pathological results also showed multiple atherosclerotic plaques and thrombosis in the vessel wall, resulting in multiple stenoses. It could also be inferred that the diameter of the fistula did not match the blood flow.

The majority of patients are asymptomatic at an early stage. The most common features are dyspnoea, fatigue, chest pain,palpitation and orthopnoea with ageing. CAFs terminating in low-pressure structures can cause left-to-right shunts, resulting in the phenomenon of “coronary artery stealing” [3]. The severity of symptoms depends on the number of shunt fistula and its influence on haemodynamics. Patients with severe clinical symptoms may develop heart failure, myocardial ischaemia or myocardial infarction (3%), arrhythmia, pulmonary hypertension, syncope attack, infective endocarditis or even sudden death. If CAFs terminate in the left heart, the haemodynamic changes are similar to aortic valve insufficiency. The size of the left ventricle may be normal, but the electrocardiographic evidence of left ventricular hypertrophy may indicate early overload [3].

Physical examination is very important, and a continuous murmur can be found in most patients. Our patient had a heart murmur at an early age but was misdiagnosed, so no further examination was performed. When he was admitted to the first hospital only 1 h after chest pain, the doctor diagnosed him with acute myocardial infarction by ECG and symptoms. Intravenous thrombolysis treatment was appropriate in that case. However, the treatment seemed useless because of the persistent chest pain and ST-segment elevation. In the second PCI-capable hospital, the extremely high D-dimer level (37,180 ng/mL) suggested massive thrombus dissolution, but there were still large numbers of undissolved clots. This result suggested that the total clot volume may be very large. The result confirmed that speculation, as the enormous clots filled the dilated artery.

Diagnostic methods include echocardiography, coronary computed tomography angioplasty (CCTA) and coronary artery angiography. Echocardiography can show the morphological features of fistulas but cannot provide more information about functional evaluation. CTA is better at anatomical delineation than echocardiography. Coronary artery angiography is the predominant diagnostic method for the precise diagnosis of fistulas. It can provide the most detailed anatomical information such as the origin, course, size, stenosis and drainage site. It can also provide the haemodynamic evaluation of the fistula and remains the modality of choice for defining coronary artery patterns for structure and flow. Furthermore, it can be used for therapeutic embolization with occlusive coils and devices [2].

The treatment of CAF includes conservative treatment, medication, transcatheter intervention, and surgery, etc. [2] The principle of all operations is to block the fistula and restore normal coronary circulation [3]. Transcatheter intervention includes stainless-steel coils, detachable balloons, double umbrella devices or Amplatzer occluders [5, 7], which is recommended according to the ACC/AHA 2008 guidelines for the management of adults with congenital heart disease, the closure of large CAFs and in patients with mild or moderate symptoms [8]. However, there is no consensus on the management of asymptomatic aneurysms. Moreover, guidelines for paediatric patients are still unavailable. In Martin Christmann’s study [9], they retrospectively examined 194 children with CAF, 10 of whom were treated by transcatheter closure or surgery. The median follow-up was 7 years. Two of them had complications. The author suggested that for paediatric patients, a personalized treatment plan should be developed based on a comprehensive consideration of CAF size, clinical symptoms, complications and even the experience of the treating centre.

The patient had a tortuous and complicated experience. From misdiagnosis in the childhood to intravenous thrombolysis treatment at a local hospital, he then underwent coronary angiography at a regional hospital and complex surgery at a national centre for cardiovascular disease. Numerous case reports and reviews have been published on coronary artery fistulas. To our knowledge, this was the first time such a giant CAF has been seen. Similar to other cases, there was no significant relationship between the clinical symptoms and the vessel diameter of the CAF.

Complete diagnosis and assessment of CAF requires not only careful physical examination but also detailed evaluation of the patient’s history, symptoms, echocardiographic or CTA data, haemodynamic effects, fistula size, location and coronary artery conditions. Therefore, we can select a suitable therapy. Surgical closure of the CAF is a safe choice for patients with symptoms. Long-term follow-up is also necessary to evaluate the management and late outcomes.

## Data Availability

Not applicable.

## Abbreviations

CAF: Coronary artery fistula
ECG: Electrocardiogram
PCI: Percutaneous coronary intervention
LM: Left main
RCA: Right coronary artery
mLAD: Middle left anterior descending
LCX: Left circumflex artery
CCTA: Coronary computed tomography angioplasty

## References

[1] Smettei OA, Abazid RM (2015). A rare case of coronary artery fistula presented with acute myocardial infarction [J]. Avicenna J Med.

[2] Challoumas D (2014). Coronary Arteriovenous fistulae: a review [J]. Int J Angiol.

[3] Jha NK, Kiraly L, Shah N, et al. Congenital aneurysmal right coronary artery with a fistula to the left atrium in an adult [J]. J Cardiothorac Surg. 2019;(1):14. 10.1186/s13019-019-0854-6 PMC6367818 30736865

[4] Buccheri Dario, Chirco Paola Rosa, Geraci Salvatore, Caramanno Giuseppe, Cortese Bernardo (2018). Coronary Artery Fistulae: Anatomy, Diagnosis and Management Strategies. Heart, Lung and Circulation.

[5] Loukas M, Germain AS, Gabriel A, John A, Tubbs RS, Spicer D (2015). Coronary artery fistula: a review. Cardiovasc Pathol.

[6] Atry T, Bicer M (2009). Coronary arteriovenous fistulas in the adults: Natural history and management strategies. J Cardiothorac Surg.

[7] Said SM, Burchart HM, Schaff HV, Conolly HM, Phillips SD, Suri RM, Eidem B, Rihal CS, Dearani JA (2013). Late outcome of repair of congenital coronary artery fistulas-a word of caution. J Thorac Cardiovasc Surg.

[8] Warnes CA, Williams RG, Bashore TM (2008). ACC/AHA 2008 guidelines for the Management of Adultswith Congenital Heart Disease: executive summary: a report of the American College of Cardiology/American Heart Association task force on practice guidelines (writing committee to develop guidelines for the management of adults with congenital heart disease). Circulation.

[9] Christmann M, Hoop R, Dave H (2017). Closure of coronary artery fistula in childhood: treatment techniques and long-term follow-up [J]. Clin Res Cardiol.

